# Sperm-borne small non-coding RNAs: potential functions and mechanisms as epigenetic carriers

**DOI:** 10.1186/s13578-025-01347-4

**Published:** 2025-01-17

**Authors:** Muhammad Naveed, Zhaokang Shen, Jianqiang Bao

**Affiliations:** 1 Center for Reproduction and Genetics, The First Affiliated Hospital of USTC, Division of Life Sciences and Medicine, University of Science and Technology of China, Hefei, Anhui 230001 China; 2 Center for Advanced Interdisciplinary Science and Biomedicine of IHM, Hefei National Laboratory for Physical Sciences at Microscale, Biomedical Sciences and Health Laboratory of Anhui Province, University of Science and Technology of China (USTC), Hefei, Anhui China

**Keywords:** Testis, Small non-coding RNAs (sncRNAs), Epigenetic inheritance, Embryonic development, Extracellular vesicles (EVs), Sperm

## Abstract

Over the past two decades, the study of sperm-borne small non-coding RNAs (sncRNAs) has garnered substantial growth. Once considered mere byproducts during germ cell maturation, these sncRNAs have now been recognized as crucial carriers of epigenetic information, playing a significant role in transmitting acquired traits from paternal to offspring, particularly under environmental influences. A growing body of evidence highlights the pivotal role of these sncRNAs in facilitating epigenetic inheritance across generations. However, the exact mechanisms through which these paternally supplied epigenetic carriers operate remain unclear and are under hot debate. This concise review presents the most extensive evidence to date on environmentally-responsive sperm-borne sncRNAs, encompassing brief summary of their origin, dynamics, compartmentalization, characteristics, as well as in-depth elaboration of their functional roles in epigenetic and transgenerational inheritance. Additionally, the review delves into the potential mechanisms by which sperm-delivered sncRNAs may acquire and transmit paternally acquired traits to offspring, modulating zygotic gene expression and influencing early embryonic development.

## Introduction

The discovery of sperm-borne small non-coding RNAs (sncRNAs) has significantly expanded our understanding of the molecular mechanisms involved in reproductive biology and early embryonic development. Traditionally, spermatozoa were primarily considered as vehicles for delivering the paternal genome to the oocyte, with the primary focus on the transmission of DNA. However, emerging research over the past two decades has revealed that sperm cells carry a complex repertoire of RNA molecules, challenging the conventional view of sperm as mere carriers of genetic information [1–4].

Sperm-borne RNAs include a diverse array of small non-coding RNAs (sncRNAs), messenger RNAs (mRNAs), and long non-coding RNAs (lncRNAs). Amidst them, the sncRNAs such as microRNAs (miRNAs), piwi-interacting RNAs (piRNAs), and tRNA-derived small RNAs (tsRNAs), have gained particular attention due to their potential roles in post-fertilization processes. These sncRNAs are now recognized as functional molecules with the capacity to influence gene expression, embryonic development, and even transgenerational inheritance [2, 5, 6].

Spermatogenesis is a precise, well-orchestrated process of events, that includes stem cell proliferation and differentiation, meiotic cell divisions and extreme chromatin condensation to produce haploid sperm cells [7]. The presence of sncRNAs in sperm is not entirely surprising given that RNAs are integral to many cellular processes, including the regulation of gene expression, maintenance of genome stability, and modulation of cellular responses to environmental stimuli. One of the most intriguing aspects of sperm-borne sncRNAs is their potential involvement in epigenetic inheritance [8, 9]. Epigenetic inheritance is defined as the transmission of epigenetic information (information that is independent of changes in DNA sequence) from parent to F1 generation when the signal originated in males or to F2 generation when the signal originated in females [10]. Epigenetic modifications, such as DNA methylation and histone modification, have long been known to influence gene expression without altering the underlying DNA sequence. Sperm-borne sncRNAs are thought to contribute to this process by carrying DNA sequence-independent genomic regulatory information that can be transmitted to the next generation [4]. This mechanism allows for the inheritance of acquired traits, where environmental factors experienced by the father can lead to alterations in sperm RNA profiles, which, in turn, can affect the development and phenotype of the offspring.

Despite rapid advances, the mechanisms by which sncRNAs exert their effects remain poorly understood. It is hypothesized that upon fertilization, sperm-borne sncRNAs may influence early embryonic gene expression by interacting with the maternal RNA pool or modulating the zygotic genome. Furthermore, the specific pathways through which sncRNAs mediate transgenerational inheritance are still under investigation, with ongoing research aiming to elucidate how these sncRNAs are introduced, packaged, and functionally active in the context of reproduction and development. The study of sperm-borne RNAs represents a rapidly evolving field that bridges the gap between molecular biology, reproductive science, and epigenetic inheritance. Understanding the role of these RNAs in heredity and development not only provides insights into fundamental biological processes but also has potential implications for human health, fertility, and disease prevention.

## Origin, dynamics and compartmentalization of sperm-borne sncRNAs

**Fig. 1:**
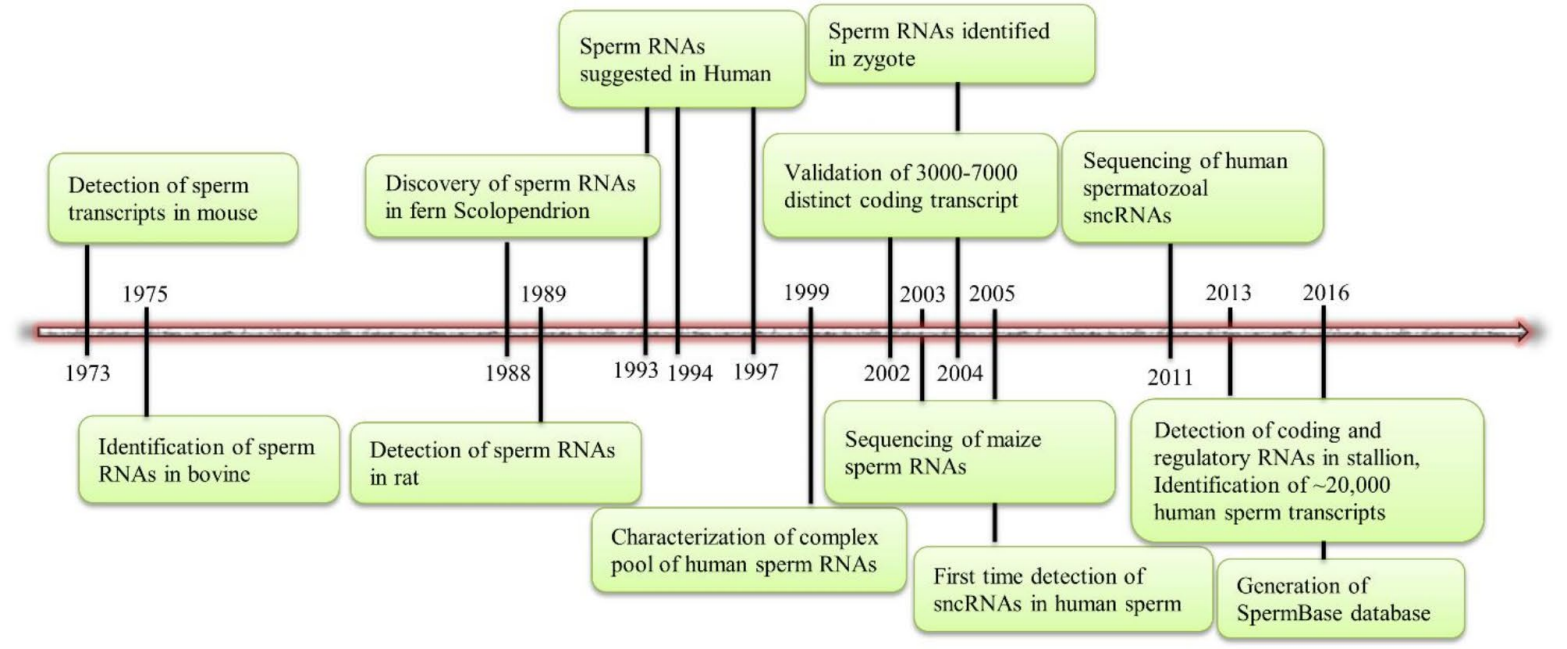
Historical timeline and perspective of spermatozoal RNAs. Sperm cells are deprived of majority of RNA molecules due to the highly compact nature of the nucleus and sparse cytoplasm. In 1973, RNAs were detected in mouse sperm for the first time [37]. Thereafter, sperm cells from multiple other species, such as bovine [38], fern Scolopendrion [39], rat [40], human [41–43] and stallion [44] have been reported with various RNA transcripts. Over the recent years, advancement in RNA-seq technologies have further characterized the complex pool of sperm transcripts in different mammalian species. For example, in 1999, Miller et al. employed cDNA cloning and sequencing techniques to characterize the intricate population of translationally quiescent human sperm RNAs for the first time [45]. According to the first global sperm transcriptome, human sperm contains roughly 3000–7000 distinct coding transcripts [46]. In 2000s, cDNA sequencing of maize sperm RNAs [47] and identification of sperm-delivered RNAs in zygotes [2] were performed. The presence and sequencing of human sperm sncRNAs were first described in 2005 and 2011, respectively [13, 48]. Sendler et al. (2013) identified over 22,000 transcripts in human sperm [14]. A SpermBase database, a database compiling sperm-borne RNAs from multiple species was generated in 2016. According to this database, miRNAs and tsRNAs are conserved small RNAs among various species and can target a large number of genes known to be vital for early development [49]

The presence of RNAs in sperm has long been a topic of intensive debate, largely due to the highly compact nature of DNA and the minimal amount of cytoplasmic remnant within the mature sperm. Testis-derived RNAs are continuously produced throughout the various stages of spermatogenesis. Compared to somatic cell types, meiotic spermatocytes and post-meiotic round spermatids exhibit extraordinarily distinctive transcriptomes [11, 12]. As transcription ceases during the late spermatid stage and most cytoplasmic contents are expelled, the RNAs detectable in sperm were initially presumed to be residual testicular RNAs or mere degradation byproducts. This debate has been significantly resolved with the identification of specific sperm transcripts across various species (Fig. 1). Advances in RNA sequencing (RNA-seq) methodologies have greatly enhanced the sensitivity, precision, and innovation in sperm RNA detection. These techniques have facilitated the identification, quantification, and characterization of a complex population of both coding and non-coding transcripts within sperm, including mRNAs, miRNAs, piRNAs, tsRNAs, endogenous small interfering RNAs (endo-siRNAs), ribosomal RNAs (rRNAs), lncRNAs, and others [4, 13, 14]. However, it remains unclear whether these sperm RNAs are actively selected or introduced in response to environmentally-induced signals.

When it comes to the dynamics of sperm-borne sncRNAs, extracellular vesicles (EVs) play a pivotal role. EVs are secreted cell-derived membrane structures that are highly heterogeneous in their origins, functions and properties [15]. Depending upon the size, three main classes of EVs can be defined. EVs, ranging from 100 nm to 1000 nm in diameter, are known as microvesicles [16] while EVs ranging in size between 1 ~ 5 μm in diameter are called apoptotic bodies [17]. The third class, best known as exosomes, is ranging from 30 ~ 150 nm [15] or even 200 nm [18] in diameter. These EVs can act as signaling vehicles and participate in cell-cell communication to maintain the body’s homeostasis through the transfer of nucleic acids, lipids, and proteins [19, 20]. EVs produced by the epithelial cells of the epididymis are known as epididymosomes, and these are the best characterized and specialized types of membrane-bounded structures, typically with 50 ~ 250 nm in size [21]. Epididymosomes perform their functions in the male reproductive tract (*i*) by eliciting a form of paracrine regulation by interacting with neighboring epithelial cells [22] (*ii*) by interacting and transiting with spermatozoa to deliver complex payload of regulatory elements that influences sperm maturation and signaling [23–25], and (*iii*) by providing protection to sperm against reactive oxygen species (ROS) [26]. Among these functions, the involvement of epididymosomes in sperm maturation by delivering regulatory elements has gained more attention and it is under intense investigation.

**Fig. 2:**
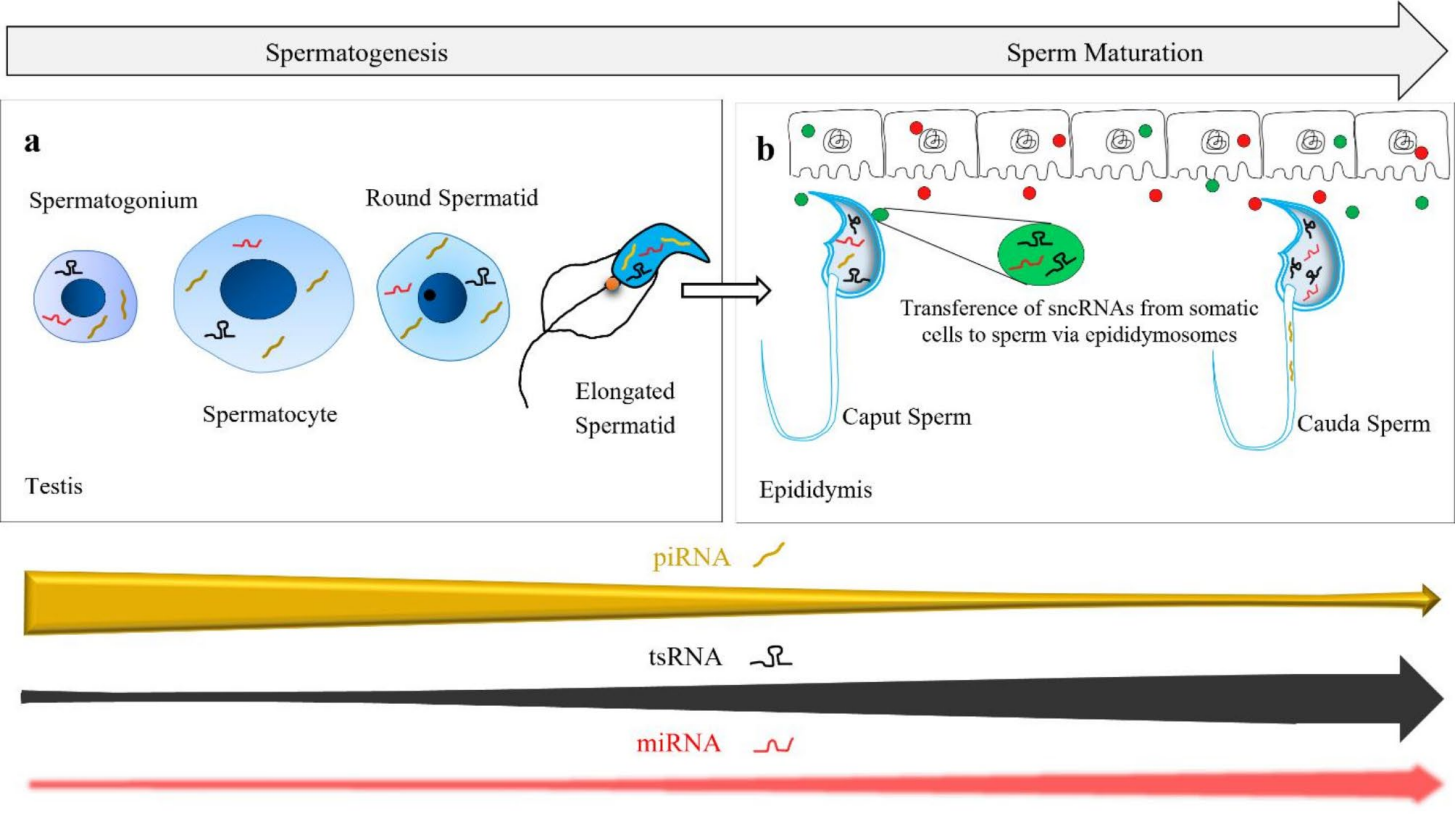
Dynamic expression patterns of the widely-studied types of sncRNAs during mammalian spermatogenesis. (**a**) Testis-derived sncRNAs: testis-derived small RNAs are continuously produced throughout the various stages of spermatogenesis. Meiotic spermatocytes and post-meiotic round spermatids exhibit extraordinarily distinctive transcriptomes. Among testis-derived sncRNAs, piRNAs are the most abundant sncRNAs while tsRNAs and miRNAs show less expression levels [11, 12]. (**b**) Soma-germline interaction and sncRNA transportation via epididymosomes: Epididymosomes are generated by the exocytosis of epididymal epithelial cells, and function as exosome-like RNA carriers, transferring the RNA repertoire to sperm during their maturation in the epididymis. Epididymosomes not only impart new sncRNAs to sperm but also likely selectively increase or enrich the copy numbers of existing sncRNAs [28, 30]. The most abundant sncRNAs in mature sperm from the cauda epididymis are tsRNAs, along with varying levels of miRNAs and piRNAs [25, 28, 29, 35, 49, 50]. The width of the arrows represents the relative abundance of specific sncRNAs throughout spermatogenesis in the testis and sperm maturation in the epididymis. Thus, sperm-borne sncRNAs are subject to dynamically spatiotemporal regulation during sperm production within the seminiferous tubules and their maturation through the epididymal tract

Mounting evidence suggest that epididymosomes are key mediators of soma-to-sperm shuttling of sncRNA repertoires [25, 27–30] (Fig. 2). These EVs have been directly implicated in the transfer of tRFs and miRNAs to the epididymal sperm. For example, Nixon has provided the first evidence for the post-testicular modification of sperm miRNA profile when sperm transit from caput to cauda epididymis. RNA sequencing data reveal the loss of 113 miRNAs and acquisition of 115 miRNAs during the sperm transition from proximal to distal epididymal segments [31]. Another study has reported the role of caput epididymosomes in delivering small RNAs to testicular spermatozoa. A dramatic switch in the RNA payload from piRNAs to tRFs has been identified when sperm transit from the testis to the epididymis [25]. In a similar vein, a 2024 study delineated the involvement of epididymosomes in the modulation of miRNA profile in sperm. This work found that a subset of miRNAs lost during spermatogenesis are partially restored during sperm epididymal transit [30]. Of note, epididymosomes not only impart new sncRNAs to sperm but also selectively increase or enrich the copy number of existing sncRNAs. For example, the copy numbers of specific miRNAs (miR-191, miR-375, miR-467a, miR-467d, and miR-467e) were expanded when sperm were incubated with epididymosomes [28]. Similarly, a markedly quantitative increase in several miRNAs, such as miR-21a, miR-29c, miR-199a, miR-200b/c, and miR-10a/b, was observed in sperm during their transit through the epididymis. In agreement with this, significant increases in miRNAs and tRFs were recorded when testicular sperm were incubated with caput epididymosomes [25, 29].

Conversely, a study conducted by Wang and his collaborators challenged these findings by showing that cytoplasmic droplets (CDs), rather than epididymosomes, are responsible for dynamic changes in small RNAs, predominantly for the tsRNAs and rsRNAs, during sperm maturation inside the epididymis. They have identified the drastic changes in sRNA composition in the caput epididymal sperm, particularly the enrichment of tsRNAs compared with testicular and cauda epididymal sperm. By applying immunofluorescent staining of the epididymal epithelial marker, sRNA-seq-based sRNA profiling, sRNA-ISH analyses and northern blot, they have confirmed that the changes resulted from sperm exchanging small RNAs with CDs rather than the epididymosomes [32]. Thus, we can conclude that epididymosomes are key players in transporting sncRNA cargos to mature sperm, however more research evidence is required to further clarify and strengthen the role of CDs in the dynamic changing of sperm sncRNA payload.

Beyond the contribution of epididymosomes and CDs for delivering sncRNAs to the sperm, the presence of EVs in the testis and their potential role in spermatogenesis has recently become a topic of interest. Recent studies have suggested that the testicular microenvironment may convey RNAs to spermatozoa through EVs [33]. A recent study reported that somatic EVs in the testis can also deliver sncRNA cargos to the elongated spermatids by crossing the blood-testis barrier (BTB) from interstitium to seminiferous tubules of testis [34] (further discussed below).

**Fig. 3:**
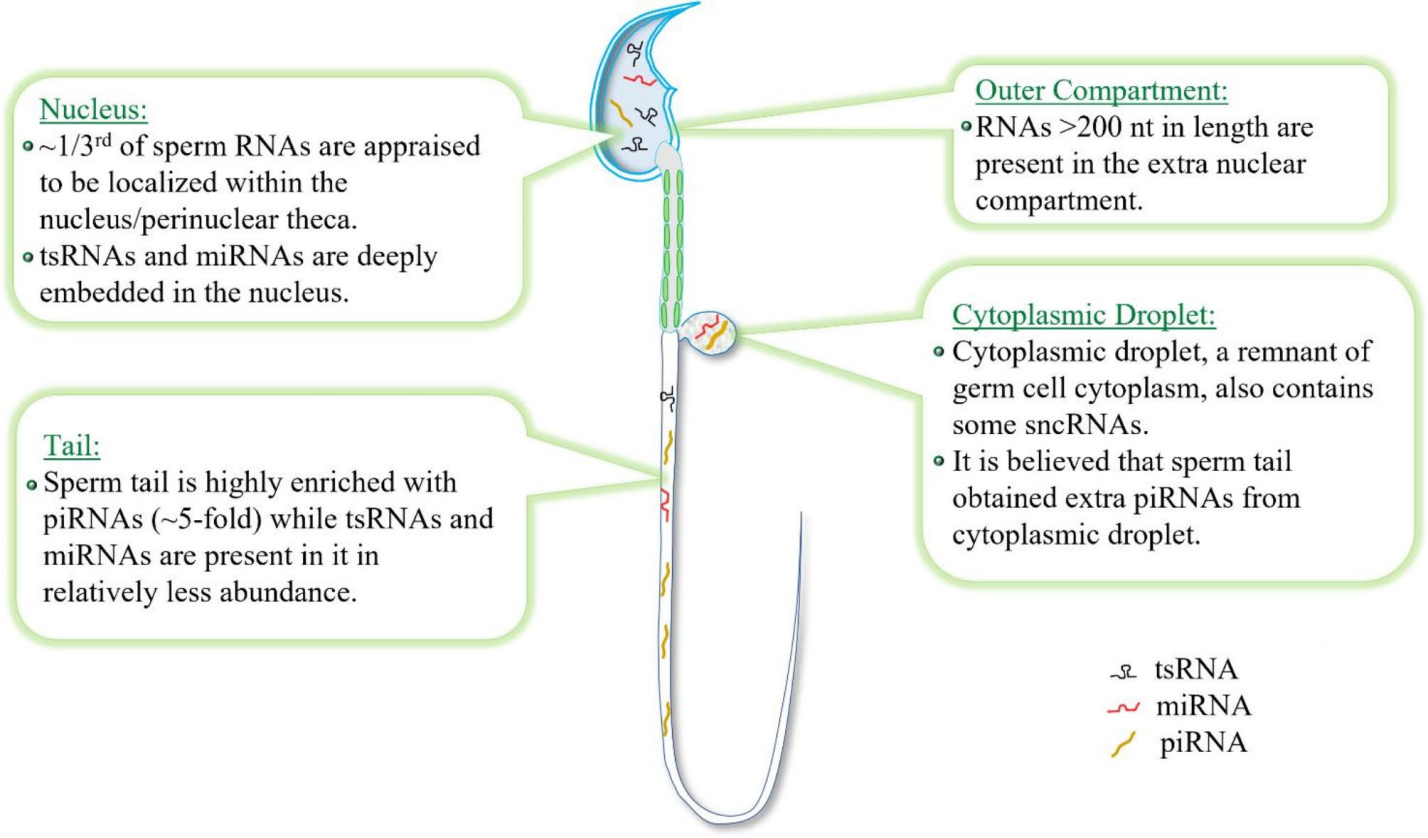
Compartmentalization of major types of sncRNAs in mature mammalian sperm. Localization of sncRNAs in mature mammalian sperm is closely associated with their developmental origins and biogenesis. Numerous studies have uncovered the presence of major types of sncRNAs in specific compartments of sperm cells, by employing techniques such as quantitative RT-PCR, in situ hybridization (ISH), microarrays, and high-throughput RNA-seq methodologies [25, 32, 35, 36, 51]. The figure lists the distribution pattern of three major types of sncRNAs (miRNAs, piRNAs and tsRNAs) within the individual region of the mature sperm cell, as indicated, based on published data. These three sncRNAs have the most significant contribution in intergenerational and transgenerational inheritance of epigenetic traits in mammals

Additionally, emerging data from multiple groups have provided compelling evidences about the localization of diverse sets of sncRNAs within the different compartments of mature mammalian sperm (Fig. 3). For instance, it has been reported that miRNAs and tsRNAs are deeply localized within the sperm nucleus [35, 36], while the sperm tail is highly enriched in piRNAs [25]. CDs, a subcellular structure transiently present in testicular and epididymal sperm, have also been identified containing some sncRNAs, particularly tsRNAs and rsRNAs [32]. Together, these studies show that sperm-borne sncRNAs are subject to dynamically spatiotemporal regulation during sperm production within the seminiferous tubules and maturation through epididymal transit.

### Types and characteristics of the most-described sperm-borne sncRNAs

High-throughput RNA-seq technology has unveiled a diverse repertoire of spermatozoal RNAs in mammals, encompassing both small non-coding RNAs (sncRNAs, < 200 nt) and long non-coding RNAs (lncRNAs, > 200 nt) [52–54]. Strikingly, although various types of sncRNAs have also been identified in oocytes [55, 56], only endogenous small interfering RNAs (endo-siRNAs) seem functional during oocyte development [57–59]. Among the most extensively studied sncRNAs in male germ cells are miRNAs, piRNAs, and tsRNAs, as briefly elucidated below (Table 1).

### miRNAs

MicroRNAs (miRNAs) are one of the most well-characterized classes of sncRNAs, constituting approximately 7% of the total sncRNA pool in the sperm of fertile men [13, 48]. These small RNAs primarily regulate gene expression by targeting the 3′ untranslated region (3′ UTR) as well as 5′ UTR of mRNAs for degradation or by repressing translation [60–62]. It is estimated that around 60% of protein-coding genes are regulated by miRNAs. High-throughput Argonaute 2 (Ago2) immunoprecipitation RNA sequencing has revealed that a single miRNA can influence gene expression by targeting multiple mRNAs in a sequence-tolerant manner, while individual mRNAs can simultaneously be targeted by multiple miRNAs [63]. miRNAs are generally highly conserved across species, although some exhibit species-specific expression [13, 35, 44, 64, 65]. Not surprisingly, miRNAs abundant in the testes are also present in mature spermatozoa [13, 66] They play key roles in numerous biological processes, including cell proliferation, apoptosis and death [67], differentiation [68], metabolism [69, 70], cell-cycle regulation [71], gametogenesis [72–75] and embryonic development [76–78]. Certain miRNAs function as transcriptional regulators by targeting other genomic regions, such as promoters and intergenic areas [79]. Notably, miRNAs are involved not only in regulating spermatogenesis within the testes [72, 73, 80], but also participate in the maturation of sperm as they transit through the epididymis [81, 82].

### piRNAs

piRNAs were identified in 2006 by four independent research teams, and represent a distinct class of sncRNAs that predominantly function in the germline. They interact with the PIWI (P-element induced wimpy testis) clade of Argonaute proteins in fruit flies, mice and humans [12, 83–86]. These small RNAs are animal species-specific and are highly expressed in the male germline, particularly in spermatocytes and spermatids [12], as well as in mature spermatozoa across various species, and are also found in mature spermatozoa of several species [13, 35, 87]. In humans, piRNAs comprise approximately 17% of the total sncRNA content [13]. Based on their precursor transcripts and timing of expression, piRNAs in mammals are classified into two major types: pre-pachytene and pachytene piRNAs. Pre-pachytene piRNAs are homologous to retroelements and are predominantly expressed in fetal and perinatal mouse testes, where they repress transposon expression to maintain germline genome integrity [88]. Pachytene piRNAs, expressed in postnatal mouse testes, primarily originate from individual piRNA clusters in intergenic regions [89]. Mechanistically, piRNAs are predominantly assumed to complex with PIWI proteins to suppress retrotransposons within the nucleus by recruiting DNA methylation and histone modification machinery to the transposon loci [88]. In the cytoplasm, they likely function in a manner similar to RNA interference (RNAi), inducing post-transcriptional silencing of target transposon mRNAs [90].

### tsRNAs

tsRNAs are the most prevalent class of regulatory sncRNAs in mammalian sperm. These RNAs arise from the cleavage of mature cytoplasmic transfer RNAs (tRNAs) or precursor tRNA molecules [35, 91]. Initially thought to be random degradation products of mature tRNAs, tsRNAs have now been recognized as products of a highly regulated process involving site-specific cleavage by endonucleases or other uncharacterized enzymes [92, 93]. These enzymes generate two major types of tsRNAs: tRNA-derived fragments (tRFs), produced from mature tRNAs, or precursors by RNase Z or Dicer, and tRNA-derived stress-induced RNAs (tiRNAs), which are 5′- or 3′-tRNA halves cleaved near the anticodon loop by angiogenin or ribonuclease Rny1 [91, 94]. Although tsRNAs have only recently been investigated, accumulating evidence suggests they regulate gene expression at the transcriptional, post-transcriptional, and translational levels, and play roles in cell proliferation, stress responses, and mRNA stability [19, 95, 96].

### Impact and role of sperm-borne sncRNAs in epigenetic inheritance

Although fully differentiated or the mature sperm in the cauda epididymis contains various types sncRNAs, their precise roles in fertilization, early embryogenesis, and offspring health remain inadequately understood. This ambiguity arises due to several factors: (1) the quantity of sperm-delivered sncRNAs at the time of fertilization is relatively low compared to the existing RNA cargos within the oocyte; (2) the oocyte itself contains similar types of abundant miRNAs that are also found in mature spermatozoa [97]; (3) the successful fertilization, pre-implantation embryo development, and production of healthy offspring in both mice and humans can be achieved by injecting round spermatids, testicular spermatozoa, or caput epididymal spermatozoa - each with distinct sncRNA payloads - into the oocytes [98–100]; and (4) the shuttling of sncRNAs between murine sperm and their CDs during sperm maturation inside the epididymis also refutes the soma-to-sperm transmission of sncRNAs [32]. These observations raise critical questions regarding the specific roles of sperm-borne sncRNAs in mediating the transmission of acquired paternal traits.

### Sperm-borne sncRNAs: environmentally-responsive epigenetic information carriers

**Fig. 4:**
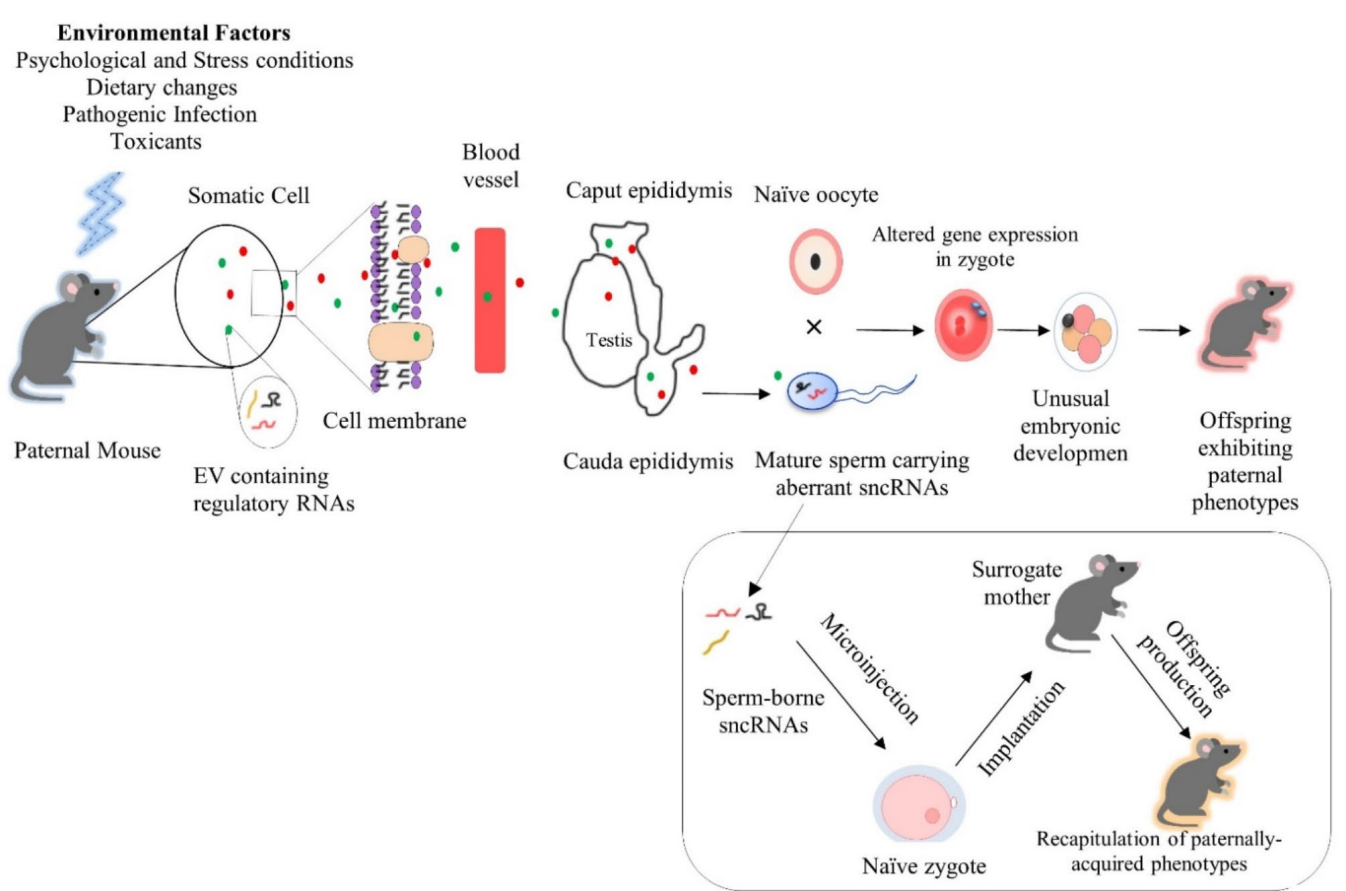
Paternal transmission of environmental factor-induced conditions to offspring via sperm-borne sncRNAs

The impact of mammalian sperm RNAs on offspring phenotypes has been highlighted by paramutation studies in mice, where purified total sperm RNAs from mutant mice were microinjected into fertilized wild-type oocytes, leading to the transmission of altered phenotypes to the offspring [101–103]. Technical advances, along with controlled studies using various model organisms, have provided exciting evidence that sperm-borne sncRNAs are environmentally responsive carriers of epigenetic information. These sncRNAs play a pivotal role in the transmission of acquired phenotypes from father to offspring, independent of Mendelian inheritance. Environmental factors such as psychological stress [104–109], dietary changes [9, 29, 110–114], toxicant exposure [115–117], pathogenic infection [118], inflammatory [119] and knockout (KO) [30] conditions can modulate the levels of sperm-borne sncRNAs. These regulated sncRNAs are significant, and in some cases, crucial contributors to fertilization, early embryonic gene expression, and the developmental programming of offspring (Table 2) (Fig. 4).

Environmental factors and stimuli are responsible for changes in various elements of nano-sized membrane-bounded somatic EVs including sncRNAs. These environmental insults compromise the integrity of the BTB or the blood-epididymis barrier, allowing the EVs to transfer sncRNA cargos (particularly miRNAs, piRNAs and tsRNAs) to immature testicular-germ cells, caput and cauda sperm cells [25, 120–122]. Sperm cells with deficient or differentially expressed sncRNA cargos can subsequently induce alterations in zygotic gene expression, leading to the atypical embryonic development and modified offspring phenotypes, mediating the intergenerational and transgenerational inheritance of epigenetic traits [107–109]. The inset illustrates that microinjection of a pool of RNAs predominantly sncRNAs, which were extracted from the sperm of males exposed to various environmental stimuli, into a naïve zygote, followed by the implantation of naïve zygote into a foster mother, results in the offspring displaying phenotypes that closely resemble to the paternally acquired traits [9, 29, 30, 105, 107, 109, 119].

### Paternal transmission of psychological and stress conditions to the offspring via sperm-borne miRNAs

Recent studies have demonstrated the pivotal role of diverse spermatozoal miRNAs in the epigenetic inheritance of psychological and stress-associated behaviors [104–109]. One of the pioneering studies by Mansuy and colleagues revealed significant alterations in the expression of several miRNAs (including up-regulation of miR-375-3p, miR-375-5p, miR-200b-3p, miR-466-5p, and miR-672-5p), along with piRNAs (notably, the down-regulation of piRNA cluster 110) in sperm from traumatized mice. These epigenetic modifications were transmitted to subsequent generations, leading to altered behaviors, hypermetabolism, and insulin hypersensitivity in the progeny [104]. In line with this finding, recent research has provided deeper insights into the mechanisms by which paternal experiences, such as early life trauma or stress, impact the sperm transcriptome and subsequently convey epigenetic information to offspring. Van Steenwyk et al. identified elevated levels of circulating factors, such as serum lipid metabolites, in exposed male mice and their offspring, which affect the sperm transcriptome and play a role in transmitting paternal allostatic load from periphery to germline to progeny. Further, in a human cohort study, a similar kind of metabolic alterations was also observed in circulation of the children that had gone through early life trauma, suggesting the conserved effects [121].

Similarly, another study reported the up-regulation of nine specific miRNAs (miR-193-5p, miR-204, miR-29c, miR-30a, miR-30c, miR-32, miR-696, miR-532-3p, and miR-698) in the sperm of mice subjected to six weeks of psychological stress [106]. Remarkably, the phenotypic effects of paternal chronic stress were recapitulated by injecting a cocktail of these nine miRNAs into embryos from non-stressed parents [105]. Benito et al. (2018) demonstrated that alterations in paternal mouse sperm RNA, particularly miRNA-212/132, in response to environmental stimuli (physical and mental exercise), contributed to the transference of enhanced cognitive abilities from parents to progeny, thereby improving cognition and synaptic plasticity in the offspring [107]. Additionally, Dickson and colleagues identified reduced levels of miR-34b, miR-34c, miR-449a, and miR-449b in the sperm of both humans and mice exposed to adverse childhood experiences and chronic social instability. They observed a reduction in several members of the miR-34/449 family in embryos at various developmental stages and in the sperm of adult offspring from stressed male mice [108], suggesting these miRNAs contribute to the transmission of non-Mendelian inheritance of behavioral phenotypes across generations.

A more recent study highlighted the direct causal role of sperm-borne miRNAs in mediating paternal transmission of behavioral disorders, such as depression-like phenotypes, to offspring. This study demonstrated that microinjection of differentially expressed miRNAs, in response to stress conditions, into naïve zygotes affected neuronal gene regulatory networks during embryonic development and facilitated the paternal-to-offspring transmission of depression-like traits through epigenetic inheritance [109]. Collectively, these findings underscore the causal relationship between specific sperm miRNAs and stress-induced phenotypes, indicating sperm miRNAs as stable, heritable epigenetic markers.

### Paternal transmission of diet-induced phenotypes to the offspring via sperm-borne sncRNAs

The studies by Fullston et al. underscore the critical role of sperm miRNAs in the transmission of diet-induced paternal phenotypes to offspring. Specifically, these studies demonstrated that a paternal high-fat diet (HFD) (21% butterfat, 34% sucrose, 17% protein) induces alterations in the miRNA profiles within both the testes and spermatozoa of F0 fathers, leading to epimutations and metabolic disturbances, such as glucose intolerance and impaired insulin sensitivity, in both male and female offspring across F1 and F2 generations. Intriguingly, obesity was observed exclusively in the female offspring. The altered miRNA cargos in the sperm of HFD-exposed fathers, including up-regulation of miR-133b-3p, miR-196a-5p, and miR-205-5p, and down-regulation of miR-340-5p, were not detected in the sperm of the offspring, suggesting that other factors beyond miRNAs are critical for the transgenerational inheritance of these metabolic phenotypes [110, 111].

Another study revealed that progeny of male mice fed a Western-like diet (high-fat, high-sugar) exhibited impaired glucose tolerance, increased body weight, and insulin resistance. Intriguingly, microinjection of RNAs from sperm or testes of diet-exposed mice into normal zygotes recapitulated these metabolic phenotypes in the offspring, reinforcing the idea that testicular germ cells harbor epigenetic information reflecting paternal pre-conceptual environmental exposures. Next-generation sequencing and qPCR analyses confirmed the deregulation of various miRNAs and piRNAs in both the testes and spermatozoa of mice fed a Western-like diet. Notably, zygotic injection of miR-19b, a significantly up-regulated miRNA in the sperm of these mice, produced offspring with increased body weight and glucose metabolism alterations, fully mirroring the paternal metabolic phenotypes [112]. These metabolic disorders persisted across subsequent generations, highlighting the active role of spermatozoal miR-19b in the transgenerational inheritance of epigenetic information (Table 2).

Chen and colleagues further elucidated the role of sperm tsRNAs in paternal RNA-mediated epigenetic inheritance of diet-induced metabolic disorders. Mice subjected to a HFD exhibited an overall increase (~ 11%) in sperm tsRNAs, and zygotic injection of these tRNA fragments led to altered expression of genes related to metabolic regulation in 8-cell embryos and blastocysts, ultimately reproducing the metabolic effects observed in naturally conceived offspring. The proposed mechanism for this non-genetic inheritance involves RNA post-transcriptional modifications, including 5-methylcytidine (m5C) and N2-methylguanosine (m2G) modifications in sperm tsRNAs.

These modifications likely confer greater stability to sperm tsRNAs as compared to their unmodified counterparts (chemically synthesized tsRNAs without RNA modifications), which degrade more rapidly in serum [123] and zygote lysates [9]. In this way, these modified sperm tsRNAs with greater stability can influence metabolic phenotypes in offspring by modulating gene expression in preimplantation embryos at both transcriptional and post-transcriptional levels [9, 33]. Overall, these results indicate that modified sperm-delivered tsRNAs act as sensitive biomarkers of environmental exposure and serve as key intergenerational carriers of diet-induced epigenetic information (Table 2). This phenomenon was partially recapitulated by another study reporting the intergenerational epigenetic inheritance of metabolic phenotypes due to altered spermatozoal sncRNA content in response to a HFD. Rats, another mammalian model, fed a HFD (21% milk fat and 34% sucrose), exhibited alterations in specific spermatozoal sncRNAs, including up-regulation of miR-let-7c-5p, tRF-Glu-CTC, tRF-Glu-TTC, piRNA-025883, and piRNA-015935, and down-regulation of miR-293-5p, miR-880-3p, and piRNA-036085. Offspring from these HFD-fed rats displayed decreased body weight, while adult female offspring had impaired glucose metabolism compared to controls. Further analysis of miR-let-7c suggested its role as a potential transgenerational carrier of HFD-induced metabolic conditions, as its expression in metabolic tissues (liver, white adipose tissue, and muscle) was altered in adult offspring, leading to disturbances in glucose and lipid metabolism [114]. Notably, several members of the let-7 miRNA family, known to regulate target genes involved in lipid and glucose metabolism, were differentially expressed in the spermatozoa of LPD-fed mice [29] and HFD-fed rats [114]. The same study found that miR-let-7c orchestrates overall metabolic phenotypes, and its deregulation in response to a HFD inhibited the translation of genes involved in glucose homeostasis and lipid metabolism, predisposing offspring to type 2 diabetes [114] (Table 2).

In another comparative study, mice fed a low-protein diet (LPD) exhibited down-regulation of several members of the let-7 miRNA family, and up-regulation of tRF-Lys-CTT, tRF-His-GTG, and various tRNA-glycine fragments (tRF-Gly-GCC, -TCC, and -CCC). Blocking a specific sperm-borne tRF-Gly-GCC resulted in the up-regulation of genes associated with murine endogenous retrovirus (MERVL), typically highly expressed in preimplantation embryos, in embryonic stem cells (ESC) and zygotes. Injection of synthetic 5’ tRF-Gly-GCC (LPD-regulated tRF) into naïve zygotes suppressed MERVL-related genes in two-cell embryos, supporting the hypothesis that specific sperm tRFs slow embryonic development after fertilization. Additionally, experiments revealed that specific genes activated during zygotic genome activation (ZGA) and associated with the totipotency program in early embryos were also regulated by tRF-Gly-GCC [29, 124]. These findings suggest that paternal LPD-altered sperm tRFs can lead to abnormal preimplantation embryonic development and, consequently, altered offspring phenotypes. Furthermore, another study identified the inhibitory role of 3’ tRFs on LTR-retrotransposons in preimplantation trophoblast stem cells [125]. Northern blot analysis confirmed the enrichment of 3’ tRFs in the epididymis and mature sperm, suggesting a potential role for sperm 3’ tRFs in regulating the endogenous retroelement MERVL in preimplantation embryos [25, 126].

A 2019 study of maternal HFD (MHFD) in mice elucidated that zygotic injection of tsRNA-enriched sperm RNA fraction (30–34 nt) from F1 male (the immediate offspring of the MHFD mother) could induce hedonic behaviours (such as overconsumption of palatable food and alcohol preference) and metabolic phenotypes (obesity and glucose metabolism) in the resulting F2 generation [113]. These findings suggest the functional specificity of the sperm tsRNAs, under the influence of MHFD, for epigenetic transgenerational inheritance. As obesity is associated with low-grade chronic inflammation [127], a recent study used an anti-inflammatory agent 5-aminosalicylic acid (5-ASA) with HFD to access the effect of this chemical on sperm tsRNA levels in sires and in the transmission of epigenetic inheritance of paternal obesity to the offspring. Results indicate that intervention with 5-ASA in HFD could decrease the levels of Glu-CTC tsRNAs in sperm cells and improve glucose tolerance in female offspring fed a chow diet [128]. Collectively, these rodent studies proved that sperm-borne sncRNAs, under the influence of different types of dietary elements, have a critical impact in the transmission of paternal metabolic phenotypes across generations, ultimately influencing progeny health (Table 2).

In addition to numerous rodent studies identifying alterations in sperm sncRNAs due to dietary factors, the first controlled study in humans also reported rapid changes in sperm sncRNAs in response to dietary perturbations. Nätt et al. (2019) investigated the influence of a two-step diet intervention on human sperm quality and sncRNA repertoire by exposing healthy young men to a week of healthy diet followed by a week of high-sugar diet. Sperm from these men exhibited increased motility after the first week, and following the second week, up-regulated levels of tsRNAs, particularly nuclear internal T-loop tsRNAs (nitRNAs). Conversely, the same nitRNAs and rRNA-derived small RNAs (rsRNAs) derived from mitochondrial DNA were down-regulated in sperm samples from obese men. Collectively, these findings demonstrate the impact of short dietary interventions on human sperm nitRNAs and rsRNAs, which are positively correlated with sperm progressive motility and negatively correlated with obesity [129], highlighting their potential as targets for studying the transmission of metabolic states from parents to offspring. Together, the emerging and extensive evidence highlights specific types of sperm-inherited miRNAs and tRFs as key players in the transmission of paternal diet-induced traits to progeny in a DNA sequence-independent manner.

### Paternal transmission of pathogenic, inflammatory and toxicant conditions to the offspring via sperm-borne sncRNAs

Tyebji et al. investigated the extensive roles of sperm small RNAs in male mice infected with the common human parasite, e.g., Toxoplasma gondii. Paternal infection with “T. gondii” induced alterations in the sperm epigenome, including significant deregulation of miRNA and piRNA levels, with 75 miRNAs up-regulated and 35 piRNAs down-regulated. Microinjection of a pool of small RNAs extracted from the sperm of “T. gondii”-infected males into control zygotes partially recapitulated the behavioral changes observed in the offspring of infected males. These findings underscore the critical role of small RNAs in the intergenerational inheritance of behavioral impairments following paternal pathogenic infection [118] (Table 2). However, the precise mechanisms through which these sperm small RNAs influence zygotic development and modulate offspring traits across multiple generations remain uncertain. Furthermore, previously it was also unclear that the pathogen itself or its associated inflammatory factors are responsible for the alteration of sperm epigenome and transmission of paternally acquired pathogenic conditions. A recent study reported the transmission of angiogenin-mediated paternal inflammation-induced metabolic disorders including glucose intolerance and obesity to the offspring via sperm tsRNAs [119]. Angiogenin is a stress-responsive RNase that mediates the cleavage of mature tRNAs within the anticodon loops, producing 5′-tsRNAs (30–35 nt) (also known as tiRNAs) and 3′-tsRNAs (40–50 nt) [91, 130]. Deletion of angiogenin prevents the inflammation-induced alteration of 5’-tsRNAs expression profile in sperm and abolishes the transmission of paternal inflammation-induced metabolic disorders to the offspring [119].

Paternal exposure to toxicant conditions such as methotrexate [116], phthalate [117] and antibiotics [131] has also been reported to cause phenotypic and metabolic disorders in the offspring by affecting the paternal sperm sncRNA landscape. Paternal exposure of dicyclohexyl phthalate (DCHP), a ubiquitous but understudied phthalate, has an adverse impact on the metabolic health of F1 and F2 offspring in mice. A novel PANDORA-seq approach has revealed the up-regulation of sperm rsRNAs and tsRNAs (tRNA-Glu-CTC-5, tRNA-Arg-CCT-2, tRNA-Arg-CCT-2 etc.) in exposed paternal mouse, supporting the notion that these altered tsRNA and rsRNA landscape carry memorized information of paternally induced phenotype and can contribute to this chemical-elicited intergenerational and transgenerational effects [117]. Similarly, paternal exposure to non-absorbable antibiotics targeting gut microbiota cause changes in offspring physiology and behaviour, by altering sperm sncRNA payload [131]. Gut microbiome have been reported as a key interface between paternal preconception environmental condition and intergenerational health in mice [132]. Masson et al. administered non-absorbable gut-microbiome depleted antibiotics to male C57BL/6J mice and identified only 8 piRNAs with differential expression (5 with up-regulation and 3 with down-regulation). And the resultant offspring (F1) of these microbiome-depleted males showed reduced body weight, shortened colon length, anxiety and depressive-like behaviours. The mechanistic understanding explicates that paternal aberrant sperm piRNAs target gene pathways entailed in nutritional deficiency and hypoxic stress that can impact the behavioural and metabolic features of the offspring [131]. Taken together, these findings demonstrate that, in addition to psychologic and diet-induced factors, pathogenic, inflammatory and toxicant conditions also have a significant impact in shaping sperm sncRNAs coding signature that, in turn, act as crucial epigenetic carriers for the transmission of traits from father to offspring (Table 2).

In summary, the studies discussed above suggest that various types of sperm-borne sncRNAs are directly modulated by specific environmental factors. For instance, psychological, trauma and stress conditions primarily affect miRNA levels, unbalanced diets (high fat, high sugar, low protein) predominantly alter tsRNAs and miRNAs, inflammatory conditions reshape tsRNAs, pathogenic conditions influence piRNAs and miRNAs and exposure to toxic compounds chiefly modify tsRNAs and rsRNAs. And these modulated sncRNAs impact critical stages of early embryonic development, potentially leading to alterations in metabolic pathways in the offspring and ultimately affecting the health of future generations.

### Potential mechanisms of sperm sncRNA-mediated epigenetic inheritance

The mechanisms by which sperm-borne sncRNAs transmit paternal preconception conditions to offspring remain a subject of intense investigation. However, recent studies have begun to illuminate how alterations in sperm sncRNA expression, initiated by external environmental insults, can be stably encoded and subsequently passed on to the next generation through non-Mendelian inheritance. This emerging evidence highlights the potential for these initial epigenetic changes to be maintained and expressed as stable phenotypes in the progeny.

### Involvement of RNA-containing EVs in epigenetic inheritance

Germline development within the seminiferous tubules of the testis is naturally insulated from interstitial cell-derived signals, largely due to the protective role of BTB. This barrier, primarily composed of Sertoli cells (SCs) with gap junctions, tight junctions, ectoplasmic specializations and desmosomes, ensures that the germline remains immune-privileged from blood-borne factors [133, 134]. However, once chromatin-condensed sperm are released from the tubules and enter the caput epididymis via the efferent ductules, they become exposed to the somatic environment and capable of responding to external signals because epididymal sperm are sensitive to the environment as compared to the developing germ cells [135]. Furthermore, epididymosomes presumably act as communicators by transferring a variety of bioactive molecules, including regulatory RNAs, from somatic cells to germ cells [25]. Interestingly, evidence suggests that EVs can enhance the long-distance delivery of functional RNAs from blood cells to neurons, particularly when the permeability of the blood-brain barrier is increased due to inflammatory responses [136]. In line with these findings, it is plausible that mental stress [137, 138] and HFD-induced obesity [120, 139], both of which are known to attenuate the BTB, could facilitate the transport of environmentally induced somatic EV cargos to immature and maturing sperm cells (Fig. 4). Notably, both psychological stress [140] and obesity [139, 141] are inherently inflammatory conditions. Cossetti’s study reported the soma-to-germline transference of RNA in mice xenografted with human tumour cells. EVs containing EGFP (enhanced green fluorescent protein) RNA can be released from xenografted human cells into the circulatory system and ultimately found in the epididymal spermatozoa [122].

Accordingly, Conine’s study further strengthens the concept of soma-to-germline shuttling of small RNAs by employing Cre/Lox genetics to generate Dgcr8 KO mice [30]. Dgcr8, a subunit of the microprocessor complex, is involved in miRNA biogenesis and processing [142]. In this study, germline- and epididymal-specific Dgcr8 KO mice were used to investigate the dynamics of sperm miRNA payload and their impact on post-fertilization. There was a loss of 27 miRNAs identified in the sperm from epididymal Dgcr8 KO mice, and this loss was responsible for altered gene expression in the embryos fertilized by this sperm. In line with this, micro-injection of these epididymal miRNAs restores the post-fertilization embryonic gene expression [30]. Hence, owing to the established role of epididymosomes in the transfer of sncRNAs to sperm, this study further explicates that these vesicles are the leading candidates to influence non-genetic information present in sperm, in the form of sncRNAs, which can subsequently regulate the development of offspring. In addition, van Steenwyk’s findings of transmission of paternal stress-induced experiences through the germline due to the elevated levels of serum lipid metabolites and altered sperm transcriptome [121], also suggest the involvement of somatic EVs in epigenetic inheritance. EVs (containing regulatory RNAs) release from the somatic cells and get into the circulating system, then cross the BTB or the blood–epididymis barrier and transfer information by affecting sperm transcriptome. Another recent study identified the presence of labelled testicular EVs within the seminiferous tubules that had been injected into the interstitial space of mouse testis. According to this study, somatic EVs can pass through the BTB from interstitium to seminiferous tubules of testis and can also deliver sncRNAs to elongated spermatids [34]. Hence, these findings support the notion that testicular EVs are permeable to the BTB and have potential contributions to the inheritable sperm sncRNA transcriptome.

Collectively, this evidence supports the notion that soma-derived factors communicate with immature germ cells in the testis via EVs or with maturing sperm via epididymosomes in the epididymal tract. This communication may enable the epigenetic transmission of acquired traits in response to environmental stimuli (Fig. 4). Although the existing evidence reveal that EVs can cross the BTB or the blood–epididymis barrier, better-designed experiments are still required to investigate the detailed mechanism and verify the ability of EVs to cross the BTB in vivo. In the sections that follow, we elaborate the mechanistic evidence of epigenetic trait transmission, as likely mediated by sperm-borne sncRNAs.

### Regulation of transcriptional cascade and reshaping of embryonic development

While the sncRNAs that respond to environmental stressors may not be abundant in sperm, and the amount of RNA injected in previous pioneering studies [9, 101, 104, 105, 112] exceeds the estimated number of sncRNAs transferred to the oocyte by a single sperm cell, the data provide compelling proof-of-principle evidence that sncRNAs possess unique properties enabling them to efficiently regulate early genomic events in the embryo. Perturbations in sncRNA levels can profoundly impact preimplantation embryonic development and offspring traits.

Under physiological conditions, mouse sperm deliver cytoplasmic information into the oocyte to influence the order of cell division and spatial patterning in developing embryos [143]. RNA-seq analysis suggests that mammalian embryos exhibit small variations in their transcriptomes at the two-cell stage due to imperfect cleavage division. These initial transcriptomic perturbations can evolve into a more defined asymmetric transcriptional pattern as the dynamic symmetry-breaking process progresses, i.e., zygotic transcriptional activation [144]. From this perspective, even slight changes in sncRNA distribution during early embryogenesis can affect the developmental process through a butterfly effect. Theoretically, environmentally-responsive sperm-borne sncRNA cargos may trigger an embryonic transcriptional cascade that influences the symmetry-breaking process, producing offspring with phenotypes that recapitulate the paternally acquired epigenetic memory.

A concrete example supporting this hypothesis is the downregulation of various genes associated with metabolic regulation observed in both early embryos and the pancreatic islets of offspring when naïve zygotic embryos were microinjected with aberrant sperm tsRNAs extracted from HFD-fed fathers [9]. This finding reinforces the notion that sperm sncRNA content influences metabolic-related genes from embryonic stages to adulthood through a transcriptional cascade. In other words, the paternally inherited sncRNA-encoded signal is maintained in early embryos and amplified into the traits observed in adults. Another example of how inherited sperm miRNAs regulate embryonic transcriptional profiles is provided by Wang and his colleagues, who found differential and aberrant expression of numerous neuronal genes in embryos generated from the sperm of males with depression-like phenotypes [109]. These genes, already implicated in the modulation of neuronal function [145–148], may be inappropriately reprogrammed and disrupted by inherited sperm miRNAs, which are typically tightly controlled and fine-tuned during early embryonic development. Essentially, inherited sperm miRNAs may induce small initial changes in core neuronal circuits during embryogenesis, leading to amplified neuronal dysfunction and the development of neuropsychiatric diseases in offspring via a butterfly effect. In this manner, altered sperm miRNAs can translate paternal environmental information and confer depression-like symptoms to offspring through epigenetic inheritance [109]. Collectively, these mechanistic findings highlight the pivotal role of sperm-borne sncRNAs in regulating transcriptional cascades during early embryonic development, and further support the concept of sperm sncRNA-mediated epigenetic inheritance.

Various studies have reported the role of sperm-borne miRNAs in the modulation of pre-implantation embryonic development in cows [149, 150], mice [5], rabbits and humans [151–154]. Such as, miR-34c, a member of the miR-34 family, has a major contribution in the first cleavage division of murine embryos [5] and in the developmental competence of embryos generated by somatic cell nuclear transfer [150, 155] and ICSI [151]. However, the mechanistic understanding of this regulation of embryonic development by miR-34c was previously missing. miR-34c is expressed in sperm and absent in oocyte [5]. A recent study clarifies that miR-34c regulates maternal mRNA and early embryonic development. According to this study, microinjection of miR-34c inhibitor in pronucleated zygotes of mice causes the upregulation expression of maternal miR-34c target mRNAs and classical maternal mRNAs. Notably, genes such as Alkbh4, Mapk14, Sp1, Sin3a, Laptm4b and Sdc1 essential for preimplantation development, also significantly downregulate after microinjection of miR-34c inhibitor [156]. In line with this finding, Liu et al. identified that more than 70% of zygotes failed to cleave after the microinjection of miR-34c inhibitor [5]. Rodger et al. reported the reduction of maternal store of mRNA transcripts such as Sirt1, Ube3a, Aars, IL6st, Srsf2, Agfg1, Ncl and Ralbp1 upto 50–75% in early zygotes due to the zygotic microinjection of specific set of sperm miRNAs. Among these transcripts, Sirt1 and Ube3a are known to play a key role in chromatin remodeling and neurodevelopmental disorders. In this way, degradation of these important maternal mRNA transcripts leads to the reprogramming of gene expression in the offspring hypothalamus with reduced hypothalamic–pituitary–adrenal (HPA) stress axis and recapitulating the stress dysregulation phenotypes in the offspring [105], as impaired maternal mRNAs prevent proper ZGA and arrest embryonic development [157]. Overall, these findings illustrate that sperm-borne miRNAs regulate the gene expression profiling of early zygotes after fertilization by interacting with maternal RNAs and play a significant role in reshaping of embryonic development and ultimately reiterate the paternal phenotypes in the offspring (Table 2).

### Sperm sncRNA modifications and epigenetic inheritance

Recent advances in RNA epigenetics have highlighted RNA post-transcriptional modifications as a crucial mechanism for transmitting paternally acquired environmental information to offspring. A notable study utilized liquid chromatography–tandem mass spectrometry (LC–MS/MS) to identify various RNA modifications in the small RNAs of mouse sperm [158], revealing a hidden layer of epigenetic regulation that adds stability to these RNAs. Specifically, a study found the significant increase in m5C and m2G in sperm tsRNAs from mice on a HFD and these modified tsRNAs preserve their functions in the oocyte after fertilization and exert their intergenerational effects [9]. RNA modifications could change the secondary structure of sperm tsRNAs in vivo and alter their targeting specificity with other RNAs, DNA or proteins, ultimately enhancing their durability and extending their half-life even after fertilization and maintain their ability to transfer epigenetic information from parents to offspring [9, 33, 159]. In line with this finding, another study reported the crucial rule of modified sperm tsRNAs by zygotic injection of 30–40 nt RNAs (predominantly 5′-tsRNAs) from angiogenin-mediated inflammatory males and non-modified synthetic 5′-tsRNAs and found that non-modified synthetic 5′-tsRNAs only partially resembled the paternal inflammation-induced metabolic disorders in offspring. Non-modified synthetic 5′-tsRNAs induces markedly less glucose intolerant yet similar obesity compared to the modified sperm RNAs, delineating that 5′- tsRNAs may exert their actions via RNA modifications dependent or independent manners [119]. These findings elucidate the complex underlying mechanism of tsRNAs-mediated transmission of paternal traits to offspring as these modifications provide more stability to tsRNAs compared to the non-modified tsRNAs.

Moreover, the enzyme DNMT2, which catalyzes the addition of m5C to specific tRNAs, has been implicated in inducing sperm tRF modifications in HFD-fed mice. The deletion of DNMT2 alters the sperm sncRNA profile, reducing the levels of modified sperm tRFs while increasing the levels of 5´-tRNA halves. This disruption ultimately prevents the transmission of HFD-induced metabolic conditions from parent to progeny [126]. In a similar context, exposure to ethanol led to the detection of modifications such as 5-methylaminomethyl-2-thiouridine and formylcytidine, which enhance RNA stability and prolong the half-life of their actions [160]. Collectively, these findings strongly suggest that various modifications in sperm-borne sncRNAs could change the structure of RNAs and alter their stability and target specificity. This can lead to the persistence of modified sperm small RNAs within the oocyte after fertilization and prolong the half-life of sperm RNA actions and play a central role in transmitting ancestrally acquired information to the offspring (Table 2).

Here, it is important to note that the aforementioned studies primarily focused on small RNA fractions with 30–40 nt in length, particularly tRFs, to identify modified nt using LC-MS/MS. One of the significant challenges in this field is mapping the entire spectrum of RNA modifications and pinpointing the specific substrates of these modifications, as LC-MS/MS alone cannot accurately determine the exact sites. To overcome this limitation, future research should employ a combination of LC-MS/MS, affinity pulldown, chemical approaches, RNA sequencing [161–163], and existing RNA modification databases [164–166]. These approaches will be instrumental in fully mapping RNA modifications in sperm and elucidating the mechanistic roles of altered post-transcriptional modifiers, particularly modified tRFs and miRNAs, in the intergenerational inheritance of acquired traits.

### Perspectives

Our comprehensive review of the sperm-borne sncRNAs highlights the current understanding of their origin, dynamics, localization, potential functions, and involvement in epigenetic inheritance, as well as the areas that require further exploration. The data presented here indicate a close association between the aberrant expression of spermatozoal sncRNAs – triggered by various environmental factors – and the intergenerational or transgenerational inheritance of epigenetic traits. The studies illustrate that the fertilizing spermatozoon is a dynamic single-cell system that delivers a complex population of sncRNAs to the egg cell upon fertilization. These sperm-delivered RNAs contribute to the molecular landscape of the embryo and influence offspring phenotypes by modulating zygotic gene expression patterns.

The consistent changes in sperm sncRNA populations from the testes to the epididymis reveal that spermatozoa acquire certain sncRNAs during testicular gametogenesis, while others are likely obtained from EVs during their transit through the epididymal tract. Furthermore, EVs play a crucial role in transferring environmentally induced epigenetic changes from somatic tissues to sperm cells via sncRNAs. However, it remains unclear whether it is possible to reverse these sperm sncRNA-mediated epigenetic modifications to prevent the transmission of unfavorable acquired traits to offspring. Additionally, it is yet to be determined whether sperm sncRNAs, as epigenetic markers, are more responsive to certain environmental factors than other epigenetic marks. The influence of one epigenetic mark over another likely depends on the developmental timing and the type of environmental stimuli. For example, mice fed a low-protein diet from birth to weaning exhibit changes in DNA methylation at ribosomal DNA (rDNA) in sperm cells [167]. In contrast, mice fed a low-protein diet starting at weaning show changes in specific small RNA levels, while rDNA methylation remains unchanged [29, 168]. This suggests that the germline epigenome is likely more vulnerable to environmental stressors during early embryonic development and primordial germ cell development, and that different sperm epigenetic factors may interact to transmit various aspects of paternally acquired traits to offspring.

The phenomenon of sperm sncRNA-mediated epigenetic inheritance has been documented in various model organisms. Some organisms, such as worms and plants, possess RNA-dependent RNA polymerase for amplifying short RNA signals [169–173], but mammals lack this enzyme. Thus, it remains unclear how sperm-delivered sncRNAs exert long-term effects on adult phenotypes. However, experimental evidence suggests that sperm-delivered sncRNAs regulate key developmental processes during the initial cell divisions post-fertilization, affecting early embryonic chromatin and DNA methylation states, which in turn can lead to long-lasting effects on offspring phenotypes in mammals. Moreover, epidemiological studies in humans have provided proof-of-concept evidence that parental exposure to trauma, stress, famine, or toxicants can influence the health of descendants [174–178]. Epigenetic inheritance of acquired traits likely plays a significant role in the etiology of complex human diseases. However, the mechanistic understanding of such transgenerational epigenetic inheritance in humans is limited due to the challenges of obtaining multigenerational cohorts, collecting cells from exposed parents, and ruling out cultural and psychological confounders. Additionally, human sperm (~ 0.3–50 fg) contains significantly less amount of RNAs compared to somatic cells (~ 3–10 pg), with approximately 200 times less RNA content overall [51, 179, 180]. This highlights the need for extensive research to unravel the nature and mechanisms of epigenetic inheritance in humans to prevent the transmission of unfavorable acquired conditions to future generations.

As described above, while a growing body of evidence supports sperm sncRNA-mediated epigenetic inheritance, our mechanistic understanding of this process remains in its infancy. Fundamental questions remain unanswered regarding the roles of sperm sncRNAs as mediators of epigenetic information. For instance, given that sperm sncRNAs likely influence early genomic events in the embryo and play a pivotal role in the intergenerational inheritance of acquired traits, how much epigenetic information is encoded within the sperm sncRNA repertoire? Do sperm-specific transcripts carry detailed epigenetic information in response to specific environmental factors, or do they convey more generalized information about overall life quality? How do sperm-borne sncRNAs transmit paternally acquired traits to future generations in humans, and what are the most concrete examples of this phenomenon? Answering these questions will be critical for understanding the influence of parental life experiences [181], accurately assessing male fertility status for pre-conception advice [180, 182], and designing therapeutic interventions to select sperm with optimal competence to improve fertility rates and embryo quality.

In conclusion, sperm-borne sncRNAs are essential for maintaining transcriptomic homeostasis during fertilization, early embryogenesis, and ZGA. Sperm with deficient or altered sncRNA cargo significantly impacts embryonic development, potentially leading to altered phenotypes in progeny. These findings suggest a novel intervention approach to improving fertility quality during IVF or ICSI procedures by injecting sperm-derived transcripts, specifically sncRNAs, into embryos. Establishing the role of sperm-borne sncRNAs in the inheritance of acquired traits is merely the first step. Sperm RNAs, either individually or in combination with other epigenetic elements, may encode information about specific acquired traits. Understanding the basic mechanisms by which this information is encoded in sperm RNAs and subsequently decoded in offspring will require advances in technologies for ‘single-cell omics’ (such as transcriptomics, ChIP-seq [183], DNA methylomics [184], and Hi-C [185]) and the design of sophisticated experiments targeting sperm and early embryonic stages. Therefore, future dedicated research using genetic and epigenetic tools will be essential to fully comprehend the hereditary flow of information as sncRNA-borne ‘codes’. We envision this review serves as a valuable summary and outlook of the extensively studied sperm-borne sncRNAs, their potential functions, and underlying mechanisms.

**Table 1:** Types, characteristics and roles of the widely-studied sperm-borne sncRNAs

Types	Length (nt)	Strand	Biogenesis	Cell types	Localization in Sperm	Generic functions	Functions in Sperm	References
**miRNA**	~18–22	Single- stranded	Nucleus and cytoplasm	Spermatogonia, spermatocyte, spermatids and mature spermatozoa	Nucleus: copious amount Tail: less amount	Transcriptional and translational regulation, cell proliferation, apoptosis, cell differentiation, metabolism, cell cycle	Sperm maturation, early embryonic development, epigenetic modification, transcriptional regulation	[13, 25, 35, 36, 186]
**piRNA**	~24–33	Single-stranded	Nucleus and cytoplasm	Spermatocytes, spermatids and mature spermatozoa	Tail	Suppression of retrotransposons within the nucleus, post-transcriptional silencing of transposon mRNAs within the cytoplasm	Pre-pachytene piRNAs: maintain germline genome integrity Pachytene piRNAs: target spermatogenesis-related mRNAs	[13, 88–90]
**tsRNA**	~29–34	Truncated fragments	Cytoplasm	Highly expressed in mature cauda epididymis sperm	Nucleus: copious amount Tail: less amount	Gene expression regulation at transcriptional, post- transcriptional and translational levels, cell proliferation, stress responses, RNA modification, protein binding, mRNA stability	Preimplantation embryonic development and transgenerational epigenetic inheritance	[19, 29, 35]

**Table 2:** Roles of environmentally responsive sperm-borne sncRNAs in epigenetic inheritance

Sperm sncRNAs	Organism	Paternal Environ mental Factors/Stimuli/Conditions	Expression	Outcome of the progeny produced through Natural mating or ICSI- procedures	Sperm RNAs micro - injection	Effects of ncRNA injection on zygote, embryo and/or offspring phenotypes	Potential Mechanisms	Reference
**Psychological and Stress Conditions**								
piRNAs and miRNAs	Mouse (C57BL/6J)	Early life trauma or psychological stress	Down-regulation: piRNA cluster 110 Up-regulation: miR-375-3p, miR-375-5p, miR-200b-3p, miR-466-5p and miR-672-5p	Depressive behaviors and metabolic alterations like hypermetabolism and insulin hypersensitivity	Total RNA	Similar spectrum of behavioral, metabolic and molecular effects observed	Increased levels of serum lipid metabolites in response to stress conditions affect the sperm transcriptome and transmit allostatic load from periphery to germline to progeny via sperm ncRNAs	[104, 121]
miRNAs	Mouse (C57/BL6:129S6/SvEvTac hybrid)	Chronic stress	Up-regulation of nine miRNAs	Stress induced phenotypes	Pool of nine miRNAs	Similar kind of stress induced phenotypes observed	A combination of this specific set of nine miRNAs causes the reduction of maternal mRNA stores in early zygotes and induces blunted HPA stress axis response and transmits stress-induced phenotypes to the offspring	[105, 106]
miRNA 212/13 2	Mouse (C57 B/6J)	Physical and mental exercise	Up- regulation: miRNA 212/132	Increased hippocampal LTP and cognitive function	Total RNA + miR212 /132 Inhibitors	Impairment of synaptic plasticity	miR212/132 may induce changes in the gene expression during embryogenesis, thereby cause subtle changes in synaptic plasticity	[107]
miR- 34/449 family	Mouse (CD-1 strain) and Human	Early life stress	Down-regulation: miR-34b, miR-34c, miR-449a and miR-449b	Stress-associated behaviors	Not studied	-	Reduced levels of miR449a and miR34c observed in different embryonic developmental stages and in the sperm of adult offspring of mice indicating the contribution of these miRNAs in the transmission of stress phenotypes up to 2 generations	[108]
miRNAs	Mouse C57BL/6J	Chronic mild, restraint and variable stress	Distinctive expression of small RNAs, miRNAs in particular	Depression-like symptoms	miRNAs	Reshaping of early embryonic transcriptional profiles and recapitulation of paternal depressive-like phenotypes	miRNAs induce preliminary tiny changes in the core neuronal circuit during embryogenesis that lead to an amplified form of neuronal dysfunction in the offspring through a butterfly effect	[109]
**Dietary Conditions**								
miRNA	Mouse (C57BL/6)	HFD	Up-regulation: miRNA-133b-3p, miRNA-196a-5p, miRNA-205-5p Down-regulation: miRNA-340-5p	Glucose intolerance and impaired insulin sensitivity in both male and female offspring, Obesity only in female offspring	Not studied	-	Modulated sperm-borne miRNAs are supposed to be delivered to the embryo during fertilization to alter the embryonic mRNA cargo, which could lead to abnormal growth of the embryo and affect metabolic phenotypes of the adult offspring in the end.	[110, 111]
miRNA-19b	Mouse (C57BL/6)	a high-fat-high-sugar diet	Up-regulation: miRNAs (miRNA-19b) and piRNAs Down-regulation: miRNAs and piRNAs	Obesity, impaired glucose tolerance and insulin resistance	Testicular RNAs and sperm miRNAs (miRNA-19b)	Increased body weight and glucose metabolic alterations	The persistence of metabolic disorders across generations due to the zygotic injection of miRNA-19b elucidating the active role of this miRNA in transgenerational epigenetic inheritance.	
tsRNAs	Mouse (C57BL/6)	HFD	Up-regulation: miRNAs and tsRNAs m^5^C and m^2^G Down-regulation: miRNAs and tsRNAs	Impaired glucose tolerance	RNA fragments 30–40 nt (predominantly tsRNAs)	Recapitulation of metabolic effects of impaired glucose tolerance	RNA modifications (m^5^C- and m^2^G-modified sperm tsRNAs) could alter the secondary structure of RNAs and increase their stability and extend their half-life as compared to the unmodified counterparts and preserve their functions in the oocyte even after fertilization. This, in turn, induces changes in the expression of metabolic regulation-related genes in early embryos which can lead to alterations in metabolic phenotypes in islets of F1 offspring	[9, 33, 159]
tsRNAs	Mouse (C57BL/6 N)	MHFD	Higher expression of tsRNAs, predominantly 5′ tRNA halves	Altered metabolic phenotypes including obesity and glucose level impairment	tsRNA-enriched RNA fraction (30–34 nt)	Stronger expression of obesogenic phenotypes and addictive-like behaviors such as overconsumption of palatable food and alcohol preference	Paternal up-regulated sperm tsRNAs, under the influence of chronic low-grade inflammation (obesity), could induce altered metabolic phenotypes in the offspring and some anti-inflammatory agents, like 5-ASA could intervene this type of epigenetic inheritance of paternal obesity by reducing the level of Glu-CTC tsRNAs in sperm cells of sires	[113, 128]
tRFs	Mouse (FVB/NJ)	LPD	Up-regulation: tRF-Gly-GCC, -TCC, -CCC, miRNAs and piRNAs Down-regulation: let-7 miRNA family and piRNAs	Reduction in the pre-implantation embryonic development	5’ tRF-Gly-GCC	Changes in the expression of genes associated with MERVL in two-cell embryos result in abnormal embryonic development	5’ tRF-Gly-GCC suppresses the transcription of MERVL-regulated genes that are highly expressed in preimplantation embryos and slows down the embryonic development by affecting placental size or function and ultimately influences offspring phenotypes as a secondary downstream effect	[29]
miRNA-let-7c	Rat (Sprague Dawley)	HFD	Up-regulation: miRNA-let7c-5p, tRF-Glu-CTC, tRF-Glu-TTC, piRNA-025883 and piRNA-015935 Down-regulation: miRNA-293-5p, miRNA-880-3p and piRNA-036085	Decreased body weight in newborn offspring and glucose intolerance in female offspring	Not studied	-	Differentially expressed miRNA-let-7c in response to HFD paradigm inhibits the translation of target genes involved in glucose homeostasis and lipid metabolism, which in turn can lead to a predisposition to type 2 diabetes	[114]
**Pathogenic**, **Inflammatory**, **Knockout and Toxicant Conditions**								
miRNAs and piRNAs	Mouse (C57BL/6J)	Infection (T. gondii)	Up-regulation: 75 miRNAs Down-regulation: 35 piRNAs	Behavioral impairments up to 2 generations	Total small RNAs	Partial recapitulation of behavioral phenotypes of naturally born offspring	-	[118]
tsRNAs	Mouse (C57BL/6)	Angiogenin-mediated inflammatory condition	Up-regulation of 5′- tsRNA-Gly-GCC, 5′-tsRNA-iMet-CAT, 5′-mt-tsRNA-Val-TAC and 5′-tsRNA-Cys-GCA	Impaired glucose tolerance and elevated fat mass	30–40 nt RNAs (predominantly 5′-tsRNAs) or a pool of synthetic tsRNAs	5′-tsRNAs from angiogenin-mediated inflammatory males induce similar metabolic disorders in the offspring while synthetic tsRNAs can partly induce metabolic phenotypes in offspring	Modifications to sperm tsRNAs provide greater stability compared to their unmodified counterparts, and these modified tsRNAs preserve their functions during fertilization and embryonic development and exert their intergenerational effects more effectively compared to the synthetic tsRNAs	[119]
piRNAs	Mouse (C57BL/6J)	Antibiotics targeting gut microbiota	Up-regulation of 5 piRNAs (mmupiR-7386, mmu-piR-10026, mmu-piR-58948, mmu-piR-30688 and mmu-piR-17932) Down-regulation of 3 piRNAs (mmu-piR-22165, mmu-piR-19528, mmupiR-54392)	Reduced body weight and altered gut morphology in F1 offspring and depressive-like behaviors in F1 females	Not studied	-	Differentially expressed paternal sperm piRNAs, in response to antibiotic cocktail administration, target gene pathways involved in nutrient scarcity and hypoxic stress and alterations to these pathways may influence metabolic features of the offspring and contribute to decrease body weight	[131]
miRNAs	Mouse (C57BL/6)	Epididymal-specific Dgcr8 KO	Down-regulation of 27 miRNAs	Epididymal-specific Dgcr8 KO males fail to produce pups via natural mating and the embryos fertilized by sperm without epididymal miRNAs exhibit altered gene expression	miRNAs purified from epididymosomes	Recapitulation of post-fertilization embryonic gene expression	Epididymosomes are the leading candidates that influence non-genetic information present in sperm by orchestrating sncRNA profile, which can subsequently regulate the development of offspring	[30]
tsRNAs and rsRNAs	Mouse (C57BL/6J)	Phthalate (DCHP)	Up-regulation of rsRNAs and tsRNAs (e.g., tRNA-Glu-CTC, tRNAArg-CCT etc.)	Exacerbated insulin resistance and impaired insulin signaling in F1 offspring and glucose intolerance in F2 females	Not studied	-	1. Paternal pre-conceptual exposure to DCHP can lead to the dysregulation of HPA axis, which may contribute to the development of metabolic disorders in the offspring 2. DCHP (a ligand of a nuclear hormonal receptor) can activate pregnane X receptor signaling pathways that in turn can lead to regulate the biogenesis of sperm tsRNAs and rsRNA	[117, 187]

## Data Availability

Not applicable.

## Abbreviations

sncRNA: Small non-coding RNA
mRNA: Messenger RNA
rRNA: Ribosomal RNA
miRNA: MicroRNA
tRNA: Transfer RNA
piRNA: Piwi-interacting RNA small
tsRNA: Transfer RNA-derived small RNA
tRFs: tRNA-derived fragments
endo-siRNA: Endogenous small interfering RNA
lncRNA: Long non-coding RNA
EVs: Extracellular vesicles
CDs: Cytoplasmic droplets
PIWI: P-element induced wimpy testis
rsRNA: rRNA-derived small RNA
RNA-seq: RNA sequencing
HFD: High-Fat Diet
MHFD: Maternal HFD
m5C: 5-methylcytidine
m2G: N2-methylguanosine
LPD: Low-protein diet
MERVL: Murine endogenous retrovirus with leucine
nitRNA: Nuclear internal T-loop tsRNA
LC–MS/MS: Liquid chromatography–tandem mass spectrometry
rDNA: Ribosomal DNA
BTB: Blood-testis barrier
ZGA: Zygotic genome activation
HPA: Hypothalamic–pituitary–adrenal
5-ASA: 5-aminosalicylic acid
SC: Sertoli cell
